# Public’s preferences for health science popularization short videos in China: a discrete choice experiment

**DOI:** 10.3389/fpubh.2023.1160629

**Published:** 2023-08-04

**Authors:** Li Xiao, Hewei Min, Yibo Wu, Jieyu Zhang, Yan Ning, Long Long, Kaixiang Jia, Weilong Jing, Xinying Sun

**Affiliations:** ^1^Publicity Division, Chinese Center for Health Education, Beijing, China; ^2^School of Public Health, Peking University, Beijing, China; ^3^School of Public Administration, Hohai University, Nanjing, China; ^4^Southern Health, Wuhan, Hubei, China; ^5^National Center for Food Safety Risk Assessment, Beijing, China

**Keywords:** discrete choice experiment, health popularization, short video, preference, health communication

## Abstract

**Background:**

Health science popularization short video disseminates health information to the public in an understandable way about health information.

**Objective:**

To investigate the preferences of Chinese residents for health science popularization short videos and provide suggestions for optimizing the production of short videos.

**Methods:**

An online survey of Chinese people was conducted using a self-administered questionnaire, and a discrete choice experiment (DCE) was used to explore the public’s preferences for health science popularization short videos.

**Results:**

A total of 618 respondents were included, of which 306 (45.51%) were male and 312 (50.49%) were female, 271 (43.85%) were aged 18–25, 239 (38.67%) were aged 26–60, and 108 (17.48%) were aged 60 and above. Whether the video is charged or not (46.891%) and the account subject (28.806%) were both considered important. The results of the DCE revealed that the participants considered video free of charge as the most significant attribute of health science popularization short videos (OR 3.433, 95% CI 3.243–3.633). Overall, participants preferred and were more willing to pay for health science popularization short videos with a hospital account subject (OR 1.192, 95% CI 1.116–1.274), with the form of graphic narration (OR 1.062, 95% CI 1.003–1.126), free of charge (OR 3.433, 95% CI 3.243–3.633), with the content that satisfies their needs (very much needed: OR 1.253, 95% CI 95% CI 1.197–1.311; generally needed: OR 1.078, 95% CI 1.029–1.129), with platform certification (OR 1.041, 95% CI 1.011–1.073), without commercial advertisements (OR 1.048, 95% CI 1.018–1.080), with simple-to-understand content (OR 1.071, 95% CI 1.040–1.104), and with video content that evokes fear or dread of illness in the viewer (OR 1.046, 95% CI 1.015–1.078).

**Conclusion:**

Participants favor free health popularization short videos, which are hospital accounts, with content that is illustrated, understandable, meets their needs, and can serve as a warning. In the future, the production of health popularization short videos should focus on improving the diversity and relevance of video content, making it as easy to understand to achieve good science popularization effects.

## 1. Introduction

The Internet has a significant impact on world development. In China, there were 1.051 billion Internet users as of June 2022, and the penetration rate were 74.4% (1). With its abilities to transcend physical and temporal borders, the Internet speeds up information dissemination, which has established itself as a crucial information channel for residents.

Short videos are one of the most widely used methods of information dissemination on the Internet. Chinese short video users reached 962 million in June 2022, an increase of 28.05 million from December 2021, making up 91.5% of the overall Internet users in the country (1). Short videos are usually under 2 min in length, with brief content, vivid images, and quick and convenient playback. These characteristics are in accordance with the public’s fragmented watching habits and have made short videos an important platform for the public to obtain information and leisure entertainment.

Health literacy is defined as “the degree to which individuals have the capacity to obtain, process, and understand basic health information and services needed to make appropriate health decisions” (2). As an important determinant and indicator of health, health literacy can influence health services utilization, health outcomes and quality of life (3, 4). However, there would be a gap between the target of 30% by *Tutorial for Outline of the Healthy China 2030 Plan* (5) and the Chinese population’s level of health literacy in 2021, which was 25.40% (6). Therefore, it is necessary to take measures to raise the health literacy level of residents. Health communication interventions have been shown to improve health literacy, increase health service utilization, and reduce risk behaviors (7–9).

Health communication was defined by Rogers as “any type of human communication whose content is concerned with health,” which includes the media agenda-setting process for health issues; media advocacy for health; scientific communication among biomedical scientists; doctor-patient communication; and, particularly, the design and evaluation of preventive health communication campaigns (10). As a type of health communication, health science popularization short videos disseminate health information to the public in an understandable way about healthy diet and lifestyle, vaccination, rational drug use, disease prevention, and other topics. Compared with medical books and newspapers, short videos combine text, images, and sound to make health information more interesting and understandable, which may influence viewers’ attitudes and behavior more easily (11–14). In addition, short videos make it possible to spread health information in just 1 min, which can achieve a large-scale spread among viewers, greatly enhancing the efficiency of health communication. Short videos and live streaming are becoming the main form of health science popularization in China. By the end of 2020, 73% of users had watched health science popularization short videos or live stream. The most popular health science popularization subjects are doctors, and 68% of audience tend to watch disease science popularization content, including healthy lifestyle and diet intervention, disease prevention and risk factors, and scientific disinformation refutations (15). Health science popularization short videos are helpful for popularizing, enhancing, and clarifying health information, which is especially important in current COVID-19 epidemic (13, 16).

Studies have shown that the public’s information adoption behaviors for short videos are closely related to the attributes of the short video. In terms of information sources, it has been demonstrated that the public’s perception of health information can be influenced by the source and channel credibility of Internet content (17), and that doctors are perceived to be more qualified, reliable, and professional (12). Regarding information content, highly engaging, clinically relevant stories may be persuasive to patients in changing health attitudes or behaviors (18). Additionally, informing about disease risks and vaccine efficacy may help improve public hesitation about the COVID-19 vaccine (19). For video length, it is suggested that short, animated story-based sugar intervention videos may need to be shorter than 2 min to engage young people or high-trait-reactance participants (20). However, a large number of health science popularization short videos still contain misinformation and disinformation and are excessively commercialized, which may prevent the public from effectively absorbing health information. Therefore, to increase the public’s adoption of health information, it is necessary to understand user characteristics, demands, and preferences for health science popularization short videos and improve the quality of content and form.

Discrete choice experiments (DCEs) are a quantitative research method to measure public preferences. In the DCE questionnaire, in order to determine participants’ preferences for a product or service, researchers design different choice options for individuals to choose from. DCEs combine random utility theory, consumer theory, experimental design theory, and econometric analysis, which are appropriate for analyzing the choice behavior of decision makers (21, 22). Recent years have seen the utilization of DCEs in the fields of vaccination, disease screening, treatment (23–25), and public preferences for health products and health information (26–28). Studies have shown that DCEs are able to predict choice-mimicking real-world decisions-if at least scale and preference heterogeneity are considered (29). However, there are currently few studies on public preferences for health science popularization short videos.

Therefore, in order to better improve the production of high-quality short videos, this study was conducted to explore the preferences of the Chinese population for health science popularization short videos through a discrete choice experiment.

## 2. Materials and methods

### 2.1. Research design

This study examined the preferences of short video audiences (age ≥ 18 years) for health science popularization short videos through a cross-sectional survey based on an online anonymous questionnaire. Health science popularization short videos are online short videos with content related to physical health, aiming to improve public health literacy and the ability to maintain health. The characteristics of health science popularization short videos are: (1) the content of the videos is related to health, such as how to maintain health and prevent and treat diseases; (2) the length of the videos ranges from a few seconds to 2 min; (3) with high-frequency feeds that are suitable for watching with mobile phones in a short time of leisure; and (4) the broadcasting platforms are TikTok, Kwai, and other video broadcasting applications. We identified 11 attributes of health science popularization short videos with a level of 2–5 levels through literature research, expert consultation, and pre-research. Additionally, using conjoint-related techniques, we designed 15 scenarios with randomly selected attribute levels, one fixed scenario, and three options. In each scenario, participants were asked to choose their preferred health science popularization short video. The McFadden’s conditional logit (CLOGIT) (30) was used to assess respondents’ preferences for attribute levels of the short videos, and their willingness to pay (WTP) was also calculated.

### 2.2. Participants

From July to October 2021, we conducted an online survey of the audience from the Southern Health short video account. Southern Health, the nation’s leading health IP industry platform, was formed in June 2018 and boasts more than 1,000 well-known health science and technology vloggers. It has established in-depth cooperation with more than 80 government agencies and 20 local radio and TV stations (31).

After receiving the questionnaire, participants were required to provide their informed consent and then respond by clicking on the questionnaire link. The inclusion criteria for participants were: age ≥ 18 years old; using smartphones with short video APPs; watching at least 10 health science popularization short videos; having basic reading and writing skills; no communication barriers. The exclusion criteria were: serious heart, liver, kidney, and other organ diseases or mental disorders; serious aphasia, disuse, and cognitive dysfunction; and not signing the informed consent. The study was approved by the Ethics Committee of the China Health Education Center (approval number: 2021004). A total of 1,500 questionnaires were distributed, and 643 of them were returned, of which 608 were valid, with a response rate of 42.87% and an effective rate of 94.56%.

### 2.3. Discrete choice experiment

#### 2.3.1. Selection of attributes and levels

We used the questionnaire to collect information. There were two sections to the questionnaire: the first part was socio-demographic characteristics, including gender, age, education level, and location; and the second part was the DCE with choice-based conjoint (CBC) scenario design of health science popularization short videos.

In DCE, alternatives are created at random based on various attributes and levels of the object, and these alternatives are then paired to form a choice set (scenarios). Each scenario requires participants to choose their preferred alternative, which usually have the highest randomized utility. We use DCE to investigated respondents’ preferences for health science popularization short videos. Firstly, we need to identify reasonable attributes and levels of health science popularization short videos. After the literature research, we conducted two rounds of large-scale expert consultation (each with 20 experts), three rounds of small-scale expert consultation (each with 1–2 experts), and a pilot survey (with 100 participants). The sites and methods of the pilot survey were the same as that of the formal survey, and the results were not included in this paper. Finally, we identified 11 attributes of health science popularization short videos (23): (1) account subject; (2) form; (3) free or not; (4) length of time; (5) content demand degree; (6) platform certification; (7) commercial advertising; (8) easy to understand; (9) funny; (10) cause the viewer’s fear or dread; and (11) video tips. Each attribute and its levels were shown in Table 1.

**Table 1:** The attributes and levels of health science popularization short videos.

Attributes	Levels
Account subject	Authoritative media; hospital;
	personal self-media (medical staff);
	personal self-media (non-medical staff);
	for-profit media (DXY, etc.)
Form	Personal commentary; graphic explanation; video clips; others (animations, etc.)
Free or not	Free of charge;1 CNY; 2 CNY; 3 CNY
Length of time/s	30; 60; 90; 120
Content demand degree	Not needed; generally needed; very much needed
Platform certification	Yes; no
Commercial advertising	Yes; no
Easy to understand	Yes; no
Funny	Yes; no
Cause the viewer’s fear or dread	Yes; no
Video tips^a^	One-side; two-side

a One-sided cues and two-sided cues were used to separate the video tips.

One-sided cues: Cueing only one’s own viewpoint or favorable material to the viewers; Two-sided cues: cueing both one’s own viewpoint or favorable material and the opposing viewpoint or unfavorable material.

#### 2.3.2. Design of alternatives and scenarios

**Figure 1:**
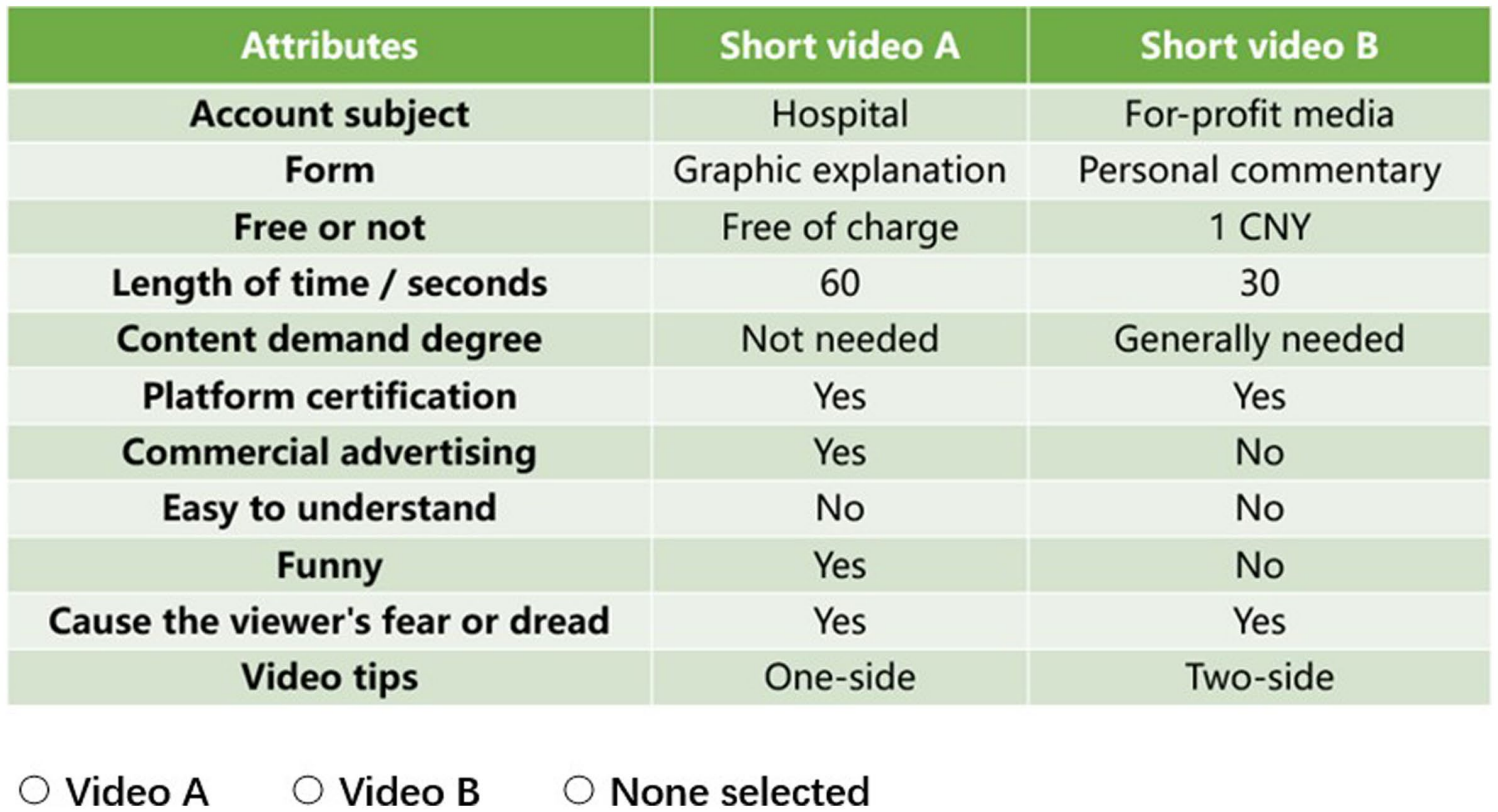
An example scenario of the DCE in the questionnaire; scenario #1/16.

Based on the identified attributes and levels, we created various alternatives which were then paired to form different scenarios for participants to choose from. Given that there were 2–5 levels for each of the 11 attributes, the possibility of 61,440 combinations (5 × 4 × 4 × 4 × 3 × 2 × 2 × 2 × 2 × 2 × 2 × 2 × 2 = 61,440) using a full factorial design was unrealistic. The ideal number of choice sets was thus generated using the fractional factorial design. Based on the principles of orthogonality, balance, and minimal overlap (23), we generated 15 random scenarios and one fixed scenario. For each scenario, we set three options (“video A”; “video B”; or “choose neither”). Participants either selected their preferred combination of health science popularization short video attributes and levels or neither of them, which could reduce the bias brought on the subjects’ forced selections (32). An example of the DCE scenario was shown in Figure 1. Using the DCE sample size formula (33) and assuming that 5% of respondents choose “choose neither,” the minimum sample size of this study is 80. A total of 618 valid samples were included in the study, which is adequate.

### 2.4. Statistical analysis

Statistical analyses were conducted using IBM SPSS Statistics (version 26.0, IBM Corporation, Armonk, NY, United States). Descriptive statistics were performed on demographic variables by frequency (composition ratio). A conditional logit model (CLOGIT) (34) was used to calculate the relative levels of attribute preferences for health science popularization short videos. Different levels of each attribute were dummy-coded, and one of the levels was chosen as the reference level. The calculation results include coefficients, *p* values, ratio ratios (OR), and 95% confidence intervals (95% CI) of OR. The coefficients indicate the value of the change in utility of an attribute level relative to the reference level, and the sign of the coefficient (positive or negative) indicates the participants’ preferred direction for a specific attribute level. Willingness to pay (WTP) was used to measure the degree to which an individual is willing to spend money in order to choose one attribute level over another (the reference attribute level). In order to illustrate the strength of respondents’ preferences for health science popularization short videos more clearly, we also calculated the WTP of participants.

## 3. Results

### 3.1. General information of the participants

A total of 618 participants were included in this study (Table 2). There were 306 (45.51%) men and 312 (50.49%) women. In terms of location, 427 (69.09%) participants lived in the city, and 191 (30.91%) participants lived in the country. There were 271 (43.85%) people aged 18–25, 239 (38.67%) people aged 26–60, and 108 (17.48%) people who were over 60 years old. Additionally, 212 (34.30%) participants had an education level of Senior high school or lower, and 406 (65.70%) participants had a college degree or higher.

**Table 2:** General characteristics of the subjects. (*n* = 618).

Items	*n* (%)
Sex	
Male	306 (45.51%)
Female	312 (50.49%)
Location	
Urban	427 (69.09%)
Rural	191 (30.91%)
Age/years	
18–25	271 (43.85%)
26–60	239 (38.67%)
>60	108 (17.48%)
Education level	
Senior high school or lower	212 (34.30%)
College or higher	406 (65.70%)

### 3.2. Percentage importance of attributes of health science popularization short videos

**Figure 2:**
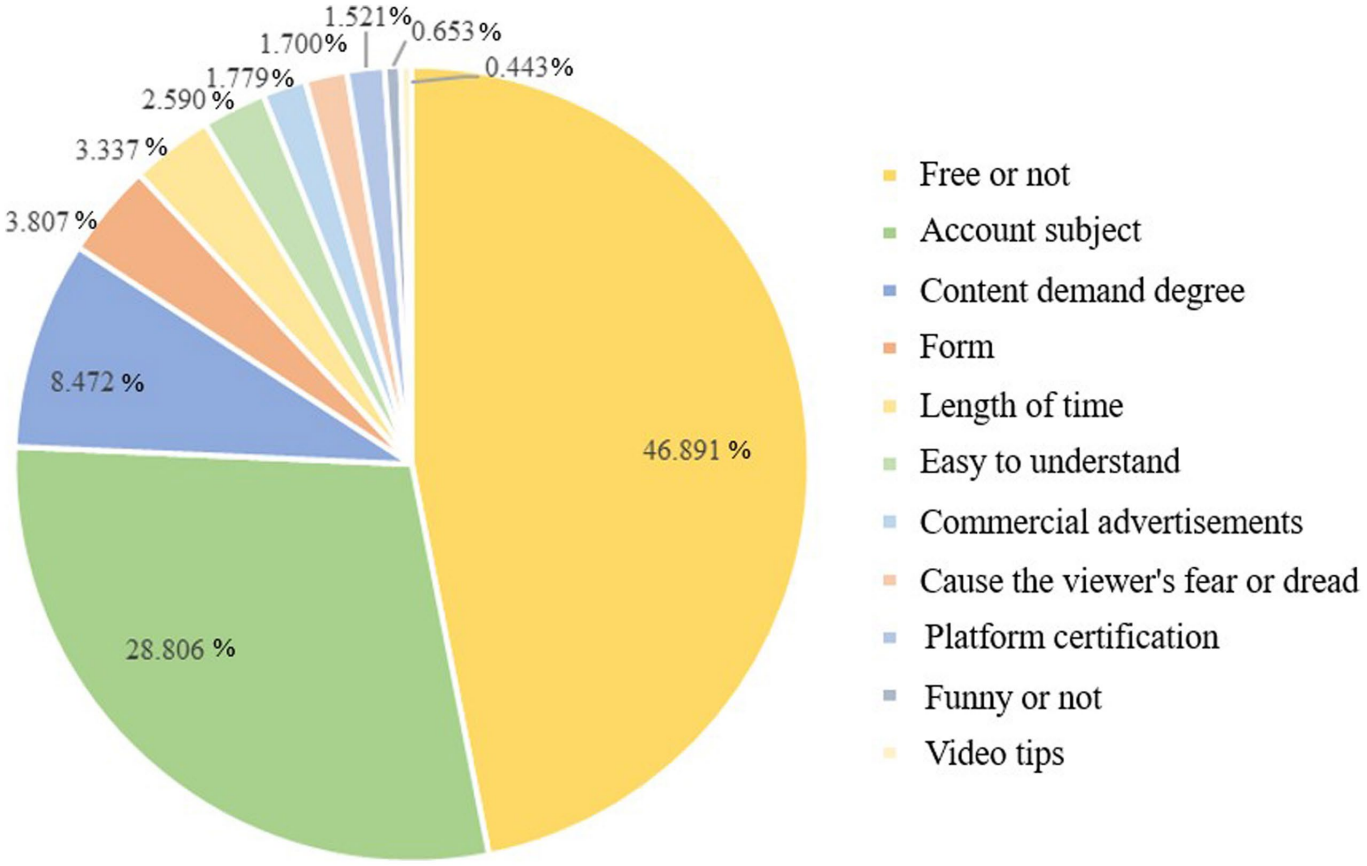
The pie chart of the percentage importance.

If the sum of the importance of all attributes is 100%, the relative importance indicates the percentage of the importance of each attribute in total attribute. To understand how each attribute influenced the participants’ overall preferences, we first assessed the relative importance of attributes of health science popularization short videos. The higher the relative importance, the more important the attribute is to participants. According to Table 3 and Figure 2, whether the video is charged or not was considered the most important attribute (46.891%), and the subject account was also considered important (28.806%). The percentage importance of the content demand degree was 8.472%, and the video format and length time were 3.807 and 3.337%, respectively. Comparatively, funny, or not and video tips were not considered as important, with importance percentages of 0.653 and 0.443%, respectively.

**Table 3:** The percentage importance of health science popularization short videos.

Attribute	Percentage importance (%)
Account subject	46.891
Form	28.806
Free or not	8.472
Length of time/seconds	3.807
Content demand degree	3.337
Platform certification	2.590
Commercial advertising	1.779
Easy to understand	1.700
Funny	1.521
Cause the viewer’s fear or dread	0.653
Video tips	0.443

### 3.3. The participants’ preferences for health science popularization short videos: results of the conditional logit model

The CLOGIT results of participants’ preferences for health science popularization short videos were shown in Table 4. According to the results, the attributes of the account subject, form, free or not, content demand degree, platform certification, easy to understand, cause the viewer’s fear or dread, and commercial advertising all influenced the participants’ preference, with free or not being the most significant factor for participants to choose health science popularization short videos. Specifically, people preferred health popularization short videos with a hospital account subject (OR 1.192, 95% CI 1.116–1.274), with the form of graphic explanation (OR 1.062, 95% CI 1.003–1.126), free of charge (OR 3.433, 95% CI 3.243–3.633), with the content that satisfy their needs (very much needed: OR 1.253, 95% CI 95% CI 1.197–1.311; generally needed: OR 1.078, 95% CI 1.029–1.129), with platform certification (OR 1.041, 95% CI 1.011–1.073), without commercial advertisements (OR 1.048, 95% CI 1.018–1.080), with easy-to-understand content (OR 1.071, 95% CI 1.040–1.104), and with video content that causes viewer’s fear or dread of illness (OR 1.046, 95% CI 1.015–1.078).

**Table 4:** CLOGIT results of participants’ preferences for health short videos.

Attributes and levels	Coefficient	*p*	OR	95%CI
Account subject				
Authoritative media^a^				
Hospital	0.176	<0.001	1.192	1.116–1.274
Personal self-media (medical staff)	−0.044	0.194	0.957	0.895–1.023
Personal self-media (non-medical staff)	−0.591	<0.001	0.554	0.515–0.595
For-profit media (DXY, etc.)	−0.528	<0.001	0.590	0.550–0.633
Form				
Personal commentary^a^				
Video clips	−0.041	0.172	0.960	0.906–1.018
Others (animations, etc.)	0.019	0.519	1.019	0.962–1.081
Graphic explanation	0.061	0.041	1.062	1.003–1.126
Free or not				
3 CNY^a^				
1 CNY	0.042	0.157	1.043	0.984–1.106
2 CNY	−0.014	0.640	0.986	0.930–1.046
Free of charge	1.233	<0.001	3.433	3.243–3.633
Length of time/seconds				
30^a^				
60	−0.031	0.298	0.970	0.915–1.027
90	0.032	0.277	1.033	0.975–1.094
120	−0.057	0.056	0.945	0.892–1.001
Content demand degree				
Not needed^a^				
Generally needed	0.075	0.002	1.078	1.029–1.129
Very much needed	0.225	<0.001	1.253	1.197–1.311
Platform certification				
No^a^				
Yes	0.040	0.008	1.041	1.011–1.073
Commercial advertisements				
Yes^a^				
No	0.047	0.002	1.048	1.018–1.080
Easy to understand				
No^a^				
Yes	0.069	<0.001	1.071	1.040–1.104
Funny				
Yes^a^				
No	0.017	0.256	1.018	0.988–1.048
Cause the viewer’s fear or dread				
No^a^				
Yes	0.045	0.003	1.046	1.015–1.078
Video tips				
One-side^a^				
Two-side	0.012	0.438	1.102	0.982–1.042

a Reference level.

### 3.4. The participants’ WTP for health science popularization short videos

Table 5 indicated the participants’ WTP for health science popularization short videos. Regarding the account subject, people were more willing to pay money to choose the hospital-based health science popularization short videos (0.154 CNY). Regarding the format of the video, people preferred short videos with graphic explanation (0.067 CNY). Meanwhile, participants were more willing to pay for 30-s video, rather than for 60-s (−0.054 CNY), 90-s (−0.046 CNY), or 120-s videos (−2.035 CNY). For the content demand degree, people were more willing to pay for videos with content met their needs (generally needed; 0.066 CNY; very much needed: 0.221 CNY). Besides, people were more likely to pay for health science popularization short videos with platform certification (0.099 CNY), free of advertisements (0.100 CNY), easy-to-understand (0.093 CNY), and that caused them fear and dread (0.078 CNY).

**Table 5:** WTP results of health science popularization short videos.

Attributes and levels	WTP (CNY)
Account subject	
Authoritative media^a^	
Hospital	−2.472^b^
Personal self-media (medical staff)	0.154
Personal self-media (non-medical staff)	−2.404
For-profit media (DXY, etc.)	−2.104
Form	
Personal commentary^a^	
Video clips	−2.019
Others (animations, etc.)	−0.303
Graphic explanation	0.067
Length of time/seconds	
30^a^	
60	−0.054
90	−0.046
120	−2.035
Content demand degree	
Not needed^a^	
Generally needed	0.066
Very much needed	0.221
Platform certification	
No^a^	
Yes	0.099
Commercial advertisements	
Yes^a^	
No	0.100
Easy to understand	
No^a^	
Yes	0.093
Funny	
Yes^a^	
No	−1.046
Cause the viewer’s fear or dread	
No^a^	
Yes	0.078
Video tips	
One-side^a^	
Two-side	−0.870

a Reference level.

b The negative value of a currency indicates that people are more willing to pay money for the reference attribute.

## 4. Discussion

### 4.1. Conclusion and recommendations

The results of this DCE revealed that Chinese audiences had specific preferences for health science popularization short videos. In general, the public prefers health science popularization short videos in which the account subject is a hospital, the presentation is in the form of a graphic explanation, it is free, the video content fulfills their demands, there is platform certification, no commercial advertisements, the video content is easy to understand, and the video content causes the viewer to fear or be afraid of the disease. In order to draw in more viewers and achieve positive science popularization effects, the production of health science popularization short videos should focus on enhancing the diversity and relevance of the video content, increasing the authority and professionalism of the content, making it simple to understand, and focusing on the public’s welfare.

### 4.2. Short videos: a new channel of health communication

Social media platforms give patients and medical professionals a new channel for communicating about health issues and health information. Moorhead et al. (35) identified seven essential functions of social media in health communication, including providing health information, responding to medical questions, fostering communication between patients and doctors, collecting data on patient experiences and opinions, using for health interventions, health promotion and health education, reducing stigma, and providing online counseling. Social media can deliver more usable, shareable, and tailored health information, and increase accessibility and widening access to health information (35). Currently, short video-based social media platforms and apps are growing in popularity among users. YouTube has become an important platform for producing and disseminating health-related videos covering topics related to chronic disease management, such as disease prevention, diagnosis, and treatment (36, 37). YouTube is also used by patients to share their personal cancer stories (38). Additionally, *TikTok Data Report 2020* revealed that as of December 2020, the number of TikTok daily active users exceeded 600 million and the average daily video searches exceeded 400 million, making it one of the most popular short video platforms (39). Short videos have significant potential for information dissemination during the COVID-19 pandemic (40). And because of their large audience size, high user stickiness, and quick and easy information dissemination, short video platforms are attractive channels for disseminating health information.

### 4.3. Audience characteristics of health science popularization short videos

In this study, the audiences of well-known Chinese health science platforms were selected, in which there were more women than men, more urban residents than rural residents, more young and middle-aged people, less older adults, and there were many participants with education level of university and above. Videos are helpful to be used to supplement or replace text when an individual’s literacy is low (41). Previous research has indicated that there is a digital divide in absorbing information and using communication technology among those with low income, education, or literacy levels, the unemployed, older adults, the disabled, the women, or the children (42, 43). Age is a major predictor of participation in social networking sites and blogging, with younger age groups reporting more frequent use (44, 45). Higher education increased the likelihood of using SNS by 13% compared to lower education (46). In addition, several studies have revealed that there are more female than male users of social networking sites (44, 45, 47). However, the limited sample size of this study makes it difficult to extrapolate differences in the distribution of health science popularization short video audiences. Therefore, future research is required to examine the digital divide, especially the socio-demographic inequalities in the viewers for health science popularization videos, and to develop strategies for bridging the split between the accessibility and adoption of health information by various demographic groups.

### 4.4. Audience preferences for account subjects of health science popularization short videos

The findings of this study indicate that the public has specific preferences for health science popularization videos. With the amount of information available on the Internet, there is a lot of unfiltered medical information that is often unscientific, misleading, or even harmful (48). According to the “5Ws” of communication (49), regarding the subject account, people like to watch health-related short videos from authoritative sources, such as doctors or hospitals, and verified by the short video platform. Aristotle’s rhetoric revealed that the image of the communicator might be “the most effective means of persuasion” by making the listener trust (50). Therefore, people trust medical professionals to communicate health information because of their authority and expertise, which allows them to act as gatekeepers of health information (48, 51, 52). Additionally, hospitals and other official health organizations have a larger professional team, which can work together to produce and disseminate health information. They can also fully utilize opinion leaders, popularize significant health issues, and promote public’s health literacy to a higher level. Platform certification can also better guarantee the authority of the science popularization short videos.

### 4.5. Audience preferences for the form of health science popularization short videos

In terms of format, this study indicated that people prefer health science popularization short videos with the presentation of graphic explanations. Studies have shown that format can serve as a motivating factor for increasing viewing time (53). Health information presented in the form of images, graphs, and charts formats is easier to understand than health information presented in text format. The combination of graphics and text not only shortens the pathway required to transform knowledge from text to imagination, but also increases the density of information per unit of time. Previous research has demonstrated that multimedia presentations, combining both graphics and text, can result in higher retention scores for health information than image-only presentations (54). Furthermore, information retrieval and learning can also benefit from information that combines text, graphics, and audio content (55, 56), which is particularly helpful for individuals with poor literacy or limited health knowledge, and who have difficulty understanding written materials on the Internet about health-related topics (57, 58). According to dual coding theory (59), image and verbal representations exist in two separate systems that complement each other to develop memory (60). The combination of images and text also enhances information recognition (61), improves motivation, facilitates the interpretation of textual content, and allows for concentration or use as a mental model (62, 63). Mayer proposed that the multimedia presentation of information will have a better impact on information adoption when it satisfies the redundancy principle (with information closely related and mutually supportive), the individual difference principle (with information meeting people with low priori knowledge), the temporal contiguity principle (with information presented simultaneously through verbal and visual materials), the spatial contiguity principle (with images and text close to each other), and the coherence principle (distracting images or text are excluded) (64). Therefore, health science popularization short videos should not only rely on the verbal presentation, but also include the visual presentation of health information, such as pictures, charts or graphs.

### 4.6. Audience preferences for the content of health science popularization short videos

For content, this study indicated that people prefer health science popularization short videos with relevant, easy-to-understand health information, or whose content can cause viewers’ fear or dread of diseases. According to the knowledge, attitude, belief and practice model (KABP model) (65), knowledge, as the primary link of health education, focuses on the effectiveness and usefulness of information. Therefore, the closer the content of health information is to the audience’s demands, the easier it is for the audience to accept, and the better the effect of information transmission. Research have shown that when the Chinese public obtains health information through the media, the most important thing is the practicality of the content, followed by the accessibility (66). In addition, while obtaining health information, the public focuses on authoritative and popular conclusions (67). Zhu et al. (68) selected the top 100 most liked short videos from Chinese provincial health committee’s account in TikTok, and found that people often followed short videos with content promoting health or disease knowledge, which are more in line with people’s need to access medical information. Content that conveys warnings might satisfy people’s fear appeals by portraying harmful information, evoking a sense of crisis and tension, and prompting preventive motivation and self-protective behavior (69). Studies have shown that viewing YouTube videos of adolescents smoking and videos suggesting that smoking increases the risk of death can effectively increase participants’ perceived prevalence and enhance beliefs about the health risks of smoking (70). In addition, users are more inclined to retweet and comment on content that appears their fears (71).

### 4.7. Audiences prefer free health science popularization short videos

Furthermore, our research revealed that individuals prefer health science popularization short videos which are free of charge and commercial advertisements. Some health information videos utilize the social reputation and user recognition of experts to attract viewers, and then implicitly or explicitly insert advertisements into their communication content, which may lead viewers to readily accept the biased opinions of the experts, abandon critical thinking, and purchase impulsively. Additionally, excessive commercialization may also constrain the creators’ thinking, resulting in flat, boring, and unconvincing short video materials and reducing the quality of content. According to the research, more than 67% of the respondents believed that the prominent problem of health science popularization short videos is the entertainment and commercialization of video content (72). Therefore, in order to promote the public’s acceptance and adoption of health information, the creation of health science popularization short videos should focus on striking a balance between public welfare and commercialism.

### 4.8. Limitations

This study has several limitations. First, because the study population was restricted to viewers of a medical short video account, it is difficult to extrapolate the findings to a larger audience. A broader population’s preferences for health science popularization short videos will need to be studied in the future in order to provide more conclusive results that are representative of the needs of the general public. Second, although the attributes and levels of health science popularization short videos were identified based on literature research and expert consultations, the scenarios presented in the questionnaire may not fully match the actual situation, and other important factors may have been overlooked. Finally, the differences and factors that influence people’s preferences for health science popularization short videos need to be further studied.

## Data availability statement

The datasets presented in this article are not readily available because the data underlying this article cannot be shared publicly due to the privacy of individuals that participated in the study. The data will be shared on reasonable request to the corresponding author. Requests to access the datasets should be directed to XS, xysun@bjmu.edu.cn.

## Ethics statement

The studies involving human participants were reviewed and approved by 2021004 (Medical ethical review committee of Chinese center for health education). The patients/participants provided their written informed consent to participate in this study.

## Author contributions

LX: study conception and design. HM: empirical analysis and writing. YW: study design and review and editing. JZ: writing. YN, LL, KJ, and WJ: acquisition of data. XS: study design and review and editing. All authors contributed to the article and approved the submitted version.

## Conflict of interest

The authors declare that the research was conducted in the absence of any commercial or financial relationships that could be construed as a potential conflict of interest.

## Publisher’s note

All claims expressed in this article are solely those of the authors and do not necessarily represent those of their affiliated organizations, or those of the publisher, the editors and the reviewers. Any product that may be evaluated in this article, or claim that may be made by its manufacturer, is not guaranteed or endorsed by the publisher.
