# Supplementary material for: Public’s preferences for health science popularization short videos in China: a discrete choice experiment
Source: Front Public Health. 2023 Aug 4;11:1160629. doi: 10.3389/fpubh.2023.1160629 (PMC10436607; doi:10.3389/fpubh.2023.1160629)
Supplement: Supplementary file 1 [file Data_Sheet_1.PDF]

A Study on Audience Preference and Satisfaction towards Health Education Short Videos

Project Name: Research on Audience's Preference and Satisfaction towards Health Education Short Videos

Project initiator: [Anonymous]

Project support unit: Southern Health Company and China Association for Health Promotion and Education

1. Invitation to participate in this study:

We would like to invite you to participate in an anonymous online survey that takes approximately 10-15 minutes. This informed consent form provides you with some information to help you decide whether to participate in this investigation. Please read carefully and if you have any questions, please raise them with the researcher.

Your participation in this study is entirely voluntary.

2.Why is this study conducted?

Understand the audience's preferences and satisfaction with health education short videos, and explore their influencing factors

3. Who will be invited to participate in this study?

The public who has watched health education short videos.

4.What are the risks of participating in this study?

Risk: Some questions in the questionnaire survey may make you feel uncomfortable or involve privacy.

5.What are the benefits of participating in this study?

You have not directly benefited, but your views on the survey content will help us better create health science short videos.

6.Is it necessary to participate in and complete this study?
Whether you participate in this study is entirely voluntary, and you can refuse or withdraw from this study at any time. We greatly hope that you can accept this survey.

7.Will my information be kept confidential?
This study will strictly manage and use data in accordance with the《National Statistical Law》, and the information and materials you fill in will be strictly confidential. The research data is for academic use only. When the research results are published, no personal information will be disclosed, nor will it have any adverse impact on you personally.

8. If I have any questions or difficulties, who should I contact?
[Anonymous]

I have read this informed consent form.

I have the opportunity to ask questions and all questions have been answered.

I understand that participating in this study is voluntary.

I can choose not to participate in this study, or withdraw at any time by notifying the researchers without discrimination or retaliation.

1.If you agree to participate in our survey, please click 'agree' below and start the survey.

○ Agree

○ Disagree (please skip to the end of the questionnaire and submit the answer sheet)

Hello, in this section, you need to answer some personal questions. Please fill in the questionnaire truthfully according to the actual situation. Thank you!

2. Your gender:

○ Male

○Female

3. Your age:

○ 18~25 ○ 26~30 ○ 31~40 ○ 41~50 ○ 51~60 ○ 60 and above

4. Your highest level of education:

○ Not receiving formal academic education

○ Primary school

○ Junior high school

○ Technical secondary school

○ High school

○ Associate degree

○ Undergraduate degree

○ Master's degree students

○ Doctoral students

5. Your marital status:

○ Unmarried

○ Married

○ Divorce

○ Widow

6. Your Permanent Residence: [Multiple Choice Question]*

○ Urban

○ Rural

7. Your family's per monthly income (CNY) *

○ ≤ 1500

○ 1501-3000

○ 3001-4500

○ 4501-6000

○ 6001-7500

○ 7501-9000

○ 9001-10500

○ 10501-12000

○ 12001-13500

○ 13501-15000

○ ≥ 15001

8. The main platform for you to watch health education short videos is: [Multiple choice questions]*

○ Tiktok

○ Kwai

○ Volcano mini video

○ Watermelon Video

○ Today's Headlines

○ WeChat video account

○ Micro vision

○ Bilibili

○ Weibo

○ Other_________________*

9. How did you come into contact with health education short videos? [Multiple choice question]*

○ Platform recommendation (based on my past browsing history)

○ Platform recommendation (unrelated to my previous browsing history)

○ Shared by friends and/or family members

○ Self-search

○ Other_________________*

10. Your preference for the content of health education short videos is [multiple choice question]*

○ Disease prevention

○ Disease treatment

○ Sports and fitness

○ Food nutrition

○ Beauty and skincare

○ Traditional Chinese Medicine Knowledge

○ Health and wellness

○ Sexual and Reproductive Health

○ Other_________________*

11. The electronic device you use to watch health education short videos is mainly:

○ Mobile phone

○ Tablets

○ Desktop computer

○ Television

○ Smart screen (such as Xiaodu)

○ Other _____*

12. The average number of consecutive health education short videos you watch each time is:

○ 1-2

○ 3-4

○ 5-6

○ 7-8

○ 9-10

○ More than 10

13. The average duration of watching health education short videos each time is:

○ 0-10 minutes

○ 10-30 minutes

○ 30-60 minutes

○ 60-120 minutes

○ over 120 minutes

14. How often do you watch health education short videos?

○ Almost every day

○ Approximately 2-4 times per week

○ About once a week

○ Approximately every two weeks

○ Approximately once a month or less

When you start watching health education short videos, you may have the following choices to watch (some options may not be in line with the actual situation). Please choose the short video you are most satisfied with in the following scenarios.

【Explanation of Terms】

One-sided Cues: Cueing only one's own viewpoint or favorable material to the viewers.

Two-sided Cues: Cueing both one's own viewpoint or favorable material and the opposing viewpoint or unfavorable material.

15. Scenario 1 (a total of 16 scenarios)

Attributes	Short Video A	Short Video B

Account subject	Personal self-media (non-medical staff)	Authoritative media
Form	Others（animations, etc.）	Personal commentary
Free or not	Free of charge	Free of charge
Length of time/ seconds	60	90
Content demand degree	Not needed	Very much needed
Platform certification	No	No
Commercial advertising	No	No
Easy to understand	No	Yes
Funny	Yes	Yes
Cause the viewer's fear or dread	Yes	No
Video tips[a]	Two-side	One-side

○ Video A ○ Video B ○ None selected

16. Scenario 2 (a total of 16 scenarios)

Attributes	Short Video A	Short Video B
Account subject	Personal self-media (medical staff)	Personal self-media (non-medical staff)
Form	Others（animations, etc.）	Video clips
Free or not	3 CNY	1 CNY
Length of time/ seconds	120	60
Content demand degree	Very much needed	Not needed
Platform certification	No	No
Commercial advertising	Yes	Yes
Easy to understand	No	Yes

Funny	No	No
Cause the viewer's fear or dread	No	No
Video tips[a]	One-side	One-side

○ Video A ○ Video B ○ None selected

17. Scenario 3 (a total of 16 scenarios)

Attributes	Short Video A	Short Video B
Account subject	Personal self-media (non-medical staff)	Personal self-media (non-medical staff)
Form	Others（animations, etc.）	Video clips
Free or not	Free of charge	1 CNY
Length of time/ seconds	30	30
Content demand degree	Very much needed	Very much needed
Platform certification	Yes	Yes
Commercial advertising	No	Yes
Easy to understand	No	Yes
Funny	No	Yes
Cause the viewer's fear or dread	No	Yes
Video tips[a]	Two-sider	One-side

○ Video A ○ Video B ○ None selected

18. Scenario 4 (a total of 16 scenarios)

Attributes	Short Video A	Short Video B
Account subject	Personal self-media (non-medical staff)	Hospital
Form	Personal commentary	Graphic explanation

Free or not	2 CNY	2 CNY
Length of time/ seconds	90	60
Content demand degree	Generally needed	Very much needed
Platform certification	Yes	Yes
Commercial advertising	Yes	No
Easy to understand	No	No
Funny	Yes	No
Cause the viewer's fear or dread	Yes	Yes
Video tips[a]	One-side	One-side

○ Video A ○ Video B ○ None selected

19. Scenario 5 (a total of 16 scenarios)

Attributes	Short Video A	Short Video B
Account subject	Personal self-media (medical staff)	Personal self-media (non-medical staff)
Form	Personal commentary	Graphic explanation
Free or not	4 CNY	4 CNY
Length of time/ seconds	60	120
Content demand degree	Very much needed	Very much needed
Platform certification	Yes	No
Commercial advertising	Yes	No
Easy to understand	Yes	Yes
Funny	Yes	Yes
Cause the viewer's fear or dread	No	Yes
Video tips[a]	Two-side	Two-side

○ Video A ○ Video B ○ None selected

20. Scenario 6 (a total of 16 scenarios)

Attributes	Short Video A	Short Video B
Account subject	Authoritative media	Personal self-media (non-medical staff)
Form	Video clips	Graphic explanation
Free or not	4 CNY	4 CNY
Length of time/ seconds	60	90
Content demand degree	Very much needed	Generally needed
Platform certification	Yes	Yes
Commercial advertising	No	No
Easy to understand	No	Yes
Funny	No	No
Cause the viewer's fear or dread	Yes	No
Video tips[a]	One-side	Two-side

○ Video A ○ Video B ○ None selected

21. Scenario 7 (a total of 16 scenarios)

Attributes	Short Video A	Short Video B
Account subject	Personal self-media (non-medical staff)	Hospital
Form	Personal commentary	Others（animations, etc.）
Free or not	2 CNY	1 CNY
Length of time/ seconds	120	90
Content demand degree	Very much needed	Very much needed
Platform certification	No	No
Commercial advertising	Yes	No

Easy to understand	No	Yes
Funny	No	Yes
Cause the viewer's fear or dread	No	No
Video tips[a]	One-side	One-side

○ Video A ○ Video B ○ None selected

22. Scenario 8 (a total of 16 scenarios)

Attributes	Short Video A	Short Video B
Account subject	Hospital	Hospital
Form	Graphic explanation	Personal commentary
Free or not	Free of charge	4 CNY
Length of time/ seconds	60	30
Content demand degree	Generally needed	Not needed
Platform certification	No	No
Commercial advertising	Yes	Yes
Easy to understand	Yes	Yes
Funny	No	No
Cause the viewer's fear or dread	No	Yes
Video tips[a]	One-side	Two-side

○ Video A ○ Video B ○ None selected

23. Scenario 9 (a total of 16 scenarios)

Attributes	Short Video A	Short Video B
Account subject	Authoritative media	Personal self-media (medical personnel)
Form	Graphic explanation	Graphic explanation
Free or not	1 CNY	Free of charge

Length of time/ seconds	120	30
Content demand degree	Not needed	Very much needed
Platform certification	Yes	Yes
Commercial advertising	Yes	Yes
Easy to understand	No	Yes
Funny	Yes	Yes
Cause the viewer's fear or dread	No	Yes
Video tips[a]	Two-side	One-side

○ Video A ○ Video B ○ None selected

24. Scenario 10 (a total of 16 scenarios)

Attributes	Short Video A	Short Video B
Account subject	Hospital	Personal self-media (medical staff)
Form	Others（animations, etc.）	Video clips
Free or not	4 CNY	Free of charge
Length of time/ seconds	90	90
Content demand degree	Not needed	Very much needed
Platform certification	Yes	No
Commercial advertising	Yes	Yes
Easy to understand	No	No
Funny	Yes	No
Cause the viewer's fear or dread	Yes	Yes
Video tips[a]	One Side Reminder	Two Side Reminder

○ Video A ○ Video B ○ None selected

25. Scenario 11 (a total of 16 scenarios)

Attributes	Short Video A	Short Video B
Account subject	Authoritative media	Hospital
Form	Others （animations, etc.）	Video clips
Free or not	2 CNY	2 CNY
Length of time/ seconds	30	120
Content demand degree	Generally needed	Very much needed
Platform certification	No	No
Commercial advertising	Yes	No
Easy to understand	Yes	Yes
Funny	No	Yes
Cause the viewer's fear or dread	Yes	Yes
Video tips[a]	Two-side	Two-side

○ Video A　　○ Video B　　○ None selected

26. Scenario 12 (a total of 16 scenarios)

Attributes	Short Video A	Short Video B
Account subject	Hospital	For-profit media (DXY, etc.)
Form	Personal commentary	Personal commentary
Free or not	1 CNY	Free of charge
Length of time/ seconds	30	120
Content demand degree	Very much needed	Not needed
Platform certification	Yes	Yes
Commercial advertising	No	No

Easy to understand	No	Yes
Funny	No	No
Cause the viewer's fear or dread	No	Yes
Video tips[a]	Two-side	One-side

○ Video A ○ Video B ○ None selected

27. Scenario 13 (a total of 16 scenarios)

Attributes	Short Video A	Short Video B
Account subject	Personal self-media (medical staff)	Hospital
Form	Video clips	Video clips
Free or not	2 CNY	Free of charge
Length of time/ seconds	90	120
Content demand degree	Not needed	Generally needed
Platform certification	Yes	Yes
Commercial advertising	No	Yes
Easy to understand	Yes	No
Funny	No	Yes
Cause the viewer's fear or dread	No	No
Video tips[a]	Two-side	Two-side

○ Video A ○ Video B ○ None selected

28. Scenario 14 (a total of 16 scenarios)

Attributes	Short Video A	Short Video B
Account subject	For-profit media (DXY, etc.)	Personal self-media (medical staff)

Form	Others（animations, etc.）	Others（animations, etc.）
Free or not	2 CNY	1 CNY
Length of time/ seconds	60	120
Content demand degree	Very much needed	Generally needed
Platform certification	Yes	Yes
Commercial advertising	Yes	No
Easy to understand	Yes	Yes
Funny	Yes	No
Cause the viewer's fear or dread	No	Yes
Video tips[a]	Two-side	One-side

○ Video A ○ Video B ○ None selected

29. Scenario 15 (a total of 16 scenarios)

Attributes	Short Video A	Short Video B
Account subject	Personal self-media (medical staff)	For-profit media (DXY, etc.)
Form	Personal commentary	Video clips
Free or not	1 CNY	4 CNY
Length of time/ seconds	60	30
Content demand degree	Generally needed	Generally needed
Platform certification	No	No
Commercial advertising	No	No
Easy to understand	No	No
Funny	Yes	Yes
Cause the viewer's fear or dread	Yes	No
Video tips[a]	Two-side	One-side

○ Video A ○ Video B ○ None selected

30. Scenario 16 (a total of 16 scenarios)

Attributes	Short Video A	Short Video B
Account subject	For-profit media (DXY, etc.)	Personal self-media (medical staff)
Form	Graphic explanation	Graphic explanation
Free or not	1 CNY	1 CNY
Length of time/ seconds	90	30
Content demand degree	Very much needed	Not needed
Platform certification	No	No
Commercial advertising	Yes	No
Easy to understand	No	No
Funny	No	Yes
Cause the viewer's fear or dread	Yes	No
Video tips[a]	Two-side	One-side

○ Video A ○ Video B ○ None selected
